# Current understanding of Alzheimer’s disease diagnosis and treatment

**DOI:** 10.12688/f1000research.14506.1

**Published:** 2018-07-31

**Authors:** Jason Weller, Andrew Budson

**Affiliations:** 1 Department of Neurology, Boston VA Hospital, 150 South Huntington Street, Jamaica Plain, MA, 02130, USA; 2 Department of Neurology, Boston University School of Medicine, 72 East Concord Street C-309, Boston, MA, USA

**Keywords:** Alzheimer's disease, dementia, amyloid, tau

## Abstract

Alzheimer’s disease is the most common cause of dementia worldwide, with the prevalence continuing to grow in part because of the aging world population. This neurodegenerative disease process is characterized classically by two hallmark pathologies: β-amyloid plaque deposition and neurofibrillary tangles of hyperphosphorylated tau. Diagnosis is based upon clinical presentation fulfilling several criteria as well as fluid and imaging biomarkers. Treatment is currently targeted toward symptomatic therapy, although trials are underway that aim to reduce the production and overall burden of pathology within the brain. Here, we discuss recent advances in our understanding of the clinical evaluation and treatment of Alzheimer’s disease, with updates regarding clinical trials still in progress.

## Background

Dementia is a clinical syndrome characterized by progressive decline in two or more cognitive domains, including memory, language, executive and visuospatial function, personality, and behavior, which causes loss of abilities to perform instrumental and/or basic activities of daily living. Alzheimer’s disease (AD) is by far the most common cause of dementia and accounts for up to 80% of all dementia diagnoses ^1^. Although the overall death rate in the United States from stroke and cardiovascular disease is decreasing, the proportion of deaths related to AD is going up, increasing by 89% between 2000 and 2014 ^2^. Direct and indirect costs for healthcare related to AD are estimated at nearly $500 billion annually ^3^. The definitive diagnosis of AD requires post-mortem evaluation of brain tissue, though cerebrospinal fluid (CSF) and positron emission tomography (PET) biomarkers combined with several relatively new clinical criteria can aid diagnosis in living patients ^4^. Current treatments available include cholinesterase inhibitors for patients with any stage of AD dementia and memantine for people with moderate-to-severe AD dementia. These medications have been shown to enhance the quality of life for both patient and caregiver when prescribed at the appropriate time during the course of illness; however, they do not change the course of illness or the rate of decline ^5^.

**Figure 1.**
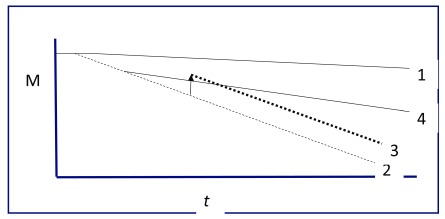
Memory and Alzheimer’s disease. Rate of decline of memory (M) over time (t, months to years). Memory declines slowly in normal aging (1). Alzheimer’s disease is marked by more rapid cognitive decline, often starting earlier in life (2). Current therapies enhance cognition without changing the rate of decline in AD (3). The anticipated effect of novel therapies is reduction in the rate of decline (4).

Clinical research is advancing toward more definitive treatment of the hallmark pathology in AD with the expectation that these therapies will attenuate the progressive cognitive decline associated with this illness ( Figure 1). This review will attempt to summarize the accepted evaluation methods and describe current and future therapies for patients with suspected AD.

## Evaluation

Building upon the original 1984 diagnostic criteria, the National Institute on Aging–Alzheimer’s Association (NIA–AA) revised the clinical criteria for the diagnosis of mild cognitive impairment (MCI) and the different stages of dementia due to AD in 2011 ^6– 8^. The use of supportive biomarker evidence (imaging, serum, and CSF) of AD pathology were included to aid in the delineation of AD from other forms of dementia as well as in the diagnosis of MCI due to AD. The Diagnostic and Statistical Manual of Mental Disorders, Fifth Edition (DSM-5) re-classified delirium, dementia, amnestic and other geriatric cognitive disorders into the more encompassing neurocognitive disorders ^9^. This change was made to better discriminate between different neurodegenerative diseases, such as AD, dementia with Lewy bodies, and frontotemporal dementia, as well as to include both major neurocognitive disorder (equivalent to dementia) and mild neurocognitive disorder (equivalent to MCI) ^4^. Finally, the newer criteria allow for the use of current and future biomarkers in the diagnosis of degenerative brain disease.

The development of non-invasive diagnostic imaging recently resulted in a test which increases the diagnostic accuracy in AD ^10^. After injection of a radiolabeled tracer agent, patients undergo a specialized PET scan that detects the deposition of amyloid-β (Aβ) peptides into plaques in the living brain. In 2012, clinicians were able to accurately diagnose the disease (later autopsy proven) using this method with up to 96% sensitivity and 100% specificity. Over the next year, this same test demonstrated similar results in patients with milder disease ^11^. Nearly a decade after researchers at the University of Pittsburgh created the first tracer, the US Food and Drug Administration approved the use of florbetapir for the detection of AD pathology. Now, the list of amyloid-specific PET ligands includes florbetaben and flutemetamol in addition to florbetapir, all of which have a similar profile ^12, 13^. However, the use of amyloid PET imaging in practice is still limited owing to its cost for most patients, as it is not covered by most insurance carriers. Currently, the majority of patients who undergo amyloid PET imaging do so as part of participation in clinical trials.

A more-invasive but less-costly evaluation involves examination of CSF for Aβ42, hyperphosphorylated tau peptide (p-tau), and total tau protein content ^14^. This method has slightly less diagnostic accuracy (85–90%), carries the risks and inconveniences involved with a lumbar puncture procedure, and often takes weeks to obtain results because of the dearth of laboratory facilities which perform the fluid analysis. However, a head-to-head comparison showed no difference in diagnostic accuracy between CSF Aβ42:p-tau ratio and amyloid PET imaging biomarkers, suggesting that the best test for individual patients depends upon availability, cost, and patient/provider preference ^15^. Less-invasive serum assays designed to detect the quantity of circulating proteins implicated in AD are currently in development and show promise. In 2017, one test discriminated among normal cognition, MCI, and dementia due to AD in a small number of patients with sensitivities and specificities of 84% and 88%, respectively ^16^. Another blood test that shows promise is the serum microRNA profile screen that demonstrated validity and reproducibility in smaller trials ^17^. With validation by future larger-scale studies, the hope is that a simple blood test may aid in the diagnosis of AD ^18^.

## Current treatment

At present, only two classes of pharmacologic therapy are available for patients with AD. The cholinesterase inhibitors donepezil, rivastigmine, and galantamine are recommended therapy for patients with mild, moderate, or severe AD dementia as well as Parkinson’s disease dementia ^19^. Memantine, which has activity as both a non-competitive N-methyl-D-aspartate receptor antagonist and a dopamine agonist, is approved for use in patients with moderate-to-severe AD (mini-mental state examination [MMSE] <15) who show difficulty with attention and alertness ^20^. For patients who choose alternative therapy, the nutraceutical huperzine A has shown benefit in both memory function and activities of daily living ^21^. However, while huperzine A is a government-approved medication outside of the US, it is not regulated by the US Food and Drug Administration and may be subject to fluctuations in potency and purity. Vitamin D deficiency was also identified as an independent risk factor for the development of dementia of any cause, and supplementation is recommended for patients in whom deficiency is diagnosed ^22^. Although many retrospective, observational studies alluded to the role of inflammation in the development of AD by showing a reduced risk of AD with the use of non-steroidal anti-inflammatory drugs, a more-thorough investigation failed to note any significant difference in cognitive performance in patients who took these medications ^23^. In the past decade, omega-3 fatty acid supplements including fish oil have received much attention owing to their cardiovascular benefits. Two recent randomized, controlled, double-blinded studies showed improvement in thinking and memory in patients with MCI who took fish oil supplements, though these studies were limited by small sample size ^24, 25^.

Finally, the management of cardiovascular risk factors contributes to overall brain health in both cerebrovascular disease and neurodegenerative disease ^26^. Recent systematic reviews found that people who adhere to the Mediterranean diet (meals consisting of fresh produce, wholegrains, olive oil, legumes, and seafood while limiting dairy and poultry products and avoiding red meat, sweets, and processed foods) have reduced risk of developing cognitive decline and AD ^27, 28^. Regular aerobic exercise, long known to prevent metabolic conditions such as diabetes mellitus and coronary artery disease, also shows preservation of function and reduces caregiver burden in patients with AD ^29^. Not only does physical exercise prevent loss of strength and agility as patients age but it also reduces neuropsychiatric symptoms and the increased care requirements associated with these issues. Recreational physical activity increases cognitive function later in life, with benefit noted regardless of age at the initiation of exercise ^30^. Less atrophy was observed in the brains of patients with genetic risk factors for AD who exercised regularly compared with those who did not, suggesting that aerobic activity prevents neurodegeneration ^31^. Although larger controlled studies are still needed to examine the long-term effects of physical activity in patients with biomarker-proven AD pathology, the inherent systemic benefits and lack of health risks should lead all healthcare providers to recommend regular exercise for their patients, regardless of cognitive function.

## Future treatment

Research into future treatments of AD involve targeting of the etiologic pathologies: neurofibrillary tangles (composed of p-tau) and senile plaques (Aβ). However, there remains debate as to which abnormality is the best target to slow or halt neurologic decline as well as how soon treatment should be initiated ^32, 33^. Another approach aims to fortify transcortical networks and enhance inter-neuronal connections in order to enhance cognitive function ^34^. From previous studies, we learned that early identification of an at-risk population and subsequent treatment in the pre-clinical stage is the approach most likely to slow or halt the progression of AD ^35^. Clinical trials are underway that aim to recruit asymptomatic patients with a genetic predisposition or biomarkers suggestive of higher risk of developing Alzheimer’s dementia, with results expected early in the next decade. The EU/US/Clinical Trials in AD Task Force in 2016 examined many of these trials in an attempt to identify the most effective measures of patient recruitment and retention, infrastructure development, and patient assessment including biomarkers and objective testing for clinical outcomes ^35^. Some of the persistent challenges identified include the timeline of recruitment and recruitment failures, difficulty in predicting success based upon prior studies for certain drugs, and the overall costs for such large-scale clinical trials. With a more cooperative effort between researchers, private and public funding, and screening of at-risk populations, a better predictor of successful clinical trials can be created.

### Anti-amyloid

According to the amyloid cascade hypothesis, toxic plaques are the earliest manifestation of disease, a statement supported by evidence of Aβ up to 20 years prior to the onset of symptoms ^36^. Researchers found in 2013 that this abnormal amyloid plaque induces the phosphorylation of tau protein, which then spreads almost infectiously via microtubule transport to neighboring neurons, leading to neuronal death ^37^. One class of medications developed using this evidence is the monoclonal antibodies (passive immunotherapy). This type of treatment involves injection of an antibody that targets abnormal Aβ and facilitates its removal from the brain. Two such monoclonal antibodies were initially developed in 2014 to remove these plaques from the brains of people with AD ^38, 39^. Neither medication improved cognitive scores in patients with mild-to-moderate disease (MMSE 16–26), leading researchers to conclude that these medications may show benefit only when administered in the early stages of MCI and mild dementia. However, a new study regarding the effect of this class of medication in patients with few to no symptoms (MMSE 20–26) but a positive amyloid PET imaging result also failed to show a significant difference in cognitive outcomes between the study group and asymptomatic controls ^40^. Studies involving similar drugs in this class are ongoing, with the goal of improving or preserving cognition in patients with MCI due to AD.

Another approach to decreasing Aβ plaque burden in the brain is the inhibition of the enzymes that produce the Aβ peptide from its precursor, amyloid precursor protein (APP). Currently, multiple drugs are in development which target β-site APP cleaving enzyme 1 (BACE1), which is thought to be essential for the production of Aβ peptides ^41^. Though previous studies of BACE1 inhibitors failed to yield meaningful results in human subjects, the novel agent verubecestat recently achieved a more than 40-fold reduction in Aβ levels in the brains of rodents and primates, and it has shown a good safety profile in early human trials ^42^. Currently, another drug is under investigation for its effect on memory and cognitive function in older patients with positive biomarkers or family history of AD, known as the EARLY study.

Researchers showed in 2014 that combination therapy with a monoclonal antibody and a BACE1 inhibitor significantly reduced the amount of Aβ in amyloid-producing mice ^43^. While there are no current trials underway utilizing this approach in humans, many experts believe that combination therapy employing both approaches to eliminate Aβ will ultimately lead to success in AD treatment ^44^.

### Anti-tau

Since p-tau appears to be the downstream pathology and is likely the direct cause of symptoms in AD, drugs to reduce the burden of this protein are also in development ^45^. Many different tau vaccines have shown both safety and efficacy in animal models ^46^, and, in one recent small study, an anti-tau drug demonstrated a good safety profile and even stimulated a positive immune response in human patients ^47^. Several other early phase trials of drugs which target the tau protein are currently underway, though results are yet to be published ^48^. Table 1 outlines the treatments and targets currently under investigation.

**Table 1.** Investigational anti-Alzheimer’s drugs.

Target	Drug	Study phase	Expected completion date	Results
β-Amyloid	CAD106	2	May 2024	
	CNP520	2	May 2024	
	BAN2401	2	November 2018	
	LY3002813 *	2	December 2020	
	Crenezumab	3	October 2022	
	Aducanumab	3	April 2022	
	UB-311	2	December 2018	
	Gantenerumab	3	November 2019	
	Solanezumab	3	Terminated May 2017	Not effective
	CT1812	2	Completed October 2016	Safe for phase 3
	Thiethylperazine	2	July 2021	
	ID1201	2	December 2018	
	NPT088	1	February 2019	
	Lu AF20513	1	October 2018	
	ABvac40	2	February 2021	
	Ponezumab	2	Completed June 2011	Not effective
	ACC-001	2	Completed February 2014	Safe for phase 3
	KHK6640	1	Completed December 2017	None yet
	GSK933776	2	Completed	Not effective
	UB-311	1	Completed	Safe for phase 2
	ABvac40	1	Completed July 2015	Safe for phase 2
BACE1	Lanabecestat	2	September 2019	
	JNJ-54861911	2	October 2022	
	Elenbecestat	3	December 2020	
	LY3202626 *	2	December 2020	
	Verubecestat	3	March 2021	
	LY450139	3	Completed April 2011	Not effective
P-tau	IONIS-MAPTRx	1, 2	February 2020	
	JNJ-63733657	1	February 2019	
	RO7105705	2	September 2022	
	ABBV-8E12	2	June 2021	
	AADvac1	2	June 2019	
	BIIB-092	2	September 2020	
	BIIB-080	1	February 2020	
	TPI-287	1	Completed May 2017	
	TRx0237	3	February 2019	
	LY3303560	1	June 2019	
APP	Posiphen	1		
RAGE	Azeliragon	3	Terminated January 2019	Not effective
Retinoid receptor	Acitretin	2	Completed February 2018	
	Bexarotene	2	Completed February 2016	

Potential treatments currently undergoing clinical investigation. APP, amyloid precursor protein; BACE1, β-site amyloid precursor protein cleaving enzyme 1; p-tau, hyperphosphorylated tau peptide; RAGE, receptor for advanced glycation end products.

*Medications under investigation as combination therapy. Source: www.clinicaltrials.gov.

### Neural circuitry

The failure of some targeted therapies toward Aβ in large-scale clinical trials has led to the hypothesis that, although the abnormal protein is implicated at the onset of AD, the progression of clinical symptoms is due to more global neural network dysfunction ^49^. Gamma oscillation, a high-frequency brainwave rhythm, is associated with inter-neuronal communication in virtually all brain networks ^50^ and may help to distinguish between true and false memories ^51^. Recently, researchers at the Massachusetts Institute of Technology found that induction of gamma-frequency oscillations led to reduced Aβ deposition and improved cognitive outcomes in an AD mouse model ^52^. This was done by using a non-invasive 40 Hz photic stimulator to entrain the desired frequency in the mouse cortex. This method is also currently in early phase trials in humans, utilizing both visual and auditory stimulation.

## Summary

As recently as 2010, the diagnosis and management of AD relied upon clinical symptom reporting that fit the pattern of memory dysfunction and loss of functional independence in multiple cognitive domains. With the reclassification system devised by the NIA–AA and DSM-5, the spectrum of AD has grown to include pre-clinical disease and MCI, helping to lay the foundation for early identification of at-risk patients. There are now a few widely available diagnostic studies that augment the clinical evaluation for a more accurate diagnosis of AD pathology, including bodily fluids and imaging studies, with good specificity.

However, the treatment options for AD remain supportive and symptomatic without attenuation of the ultimate prognosis. Medications such as cholinesterase inhibitors and memantine improve memory and alertness, respectively, without changing the life expectancy or overall progression of AD dementia. Lifestyle modifications including diet and exercise remain the only interventions with evidence showing lower AD risk and possible prevention of overall cognitive decline, and these interventions are first-line recommendations for all patients regardless of cognitive function. The pathological features associated with AD, Aβ and p-tau, are the current targets for potential treatments; however, early success in comparative studies and smaller clinical trials are thus far not reproducible in larger-scale administrations. Although limited evidence suggests that earlier identification of AD pathology will lead to better and more-definitive treatment, the results of larger-scale interventions are not yet available for review. Given the rising prevalence and mortality of AD coupled with the growing total healthcare costs, there continues to be a sense of urgency in the medical community to develop effective means for the early diagnosis and successful treatment of this progressive neurodegenerative disease.

## Abbreviations

Aβ, amyloid β; AD, Alzheimer’s disease; APP, amyloid precursor protein; BACE1, β-site amyloid precursor protein cleaving enzyme 1; CSF, cerebrospinal fluid; DSM-5, Diagnostic and Statistical Manual of Mental Disorders, Fifth Edition; MCI, mild cognitive impairment; MMSE, mini-mental state examination; NIA–AA, National Institute on Aging–Alzheimer’s Association; p-tau, hyperphosphorylated tau peptide; PET, positron emission tomography
